# Universal influenza vaccines: Shifting to better vaccines

**DOI:** 10.1016/j.vaccine.2016.03.085

**Published:** 2016-06-03

**Authors:** Francesco Berlanda Scorza, Vadim Tsvetnitsky, John J. Donnelly

**Affiliations:** PATH, 2201 Westlake Avenue Suite 200, Seattle, WA 98121, USA

**Keywords:** Influenza vaccines, Universal vaccines, Chimeric hemagglutinin, Heterosubtypic immunity

## Abstract

Influenza virus causes acute upper and lower respiratory infections and is the most likely, among known pathogens, to cause a large epidemic in humans. Influenza virus mutates rapidly, enabling it to evade natural and vaccine-induced immunity. Furthermore, influenza viruses can cross from animals to humans, generating novel, potentially pandemic strains. Currently available influenza vaccines induce a strain specific response and may be ineffective against new influenza viruses. The difficulty in predicting circulating strains has frequently resulted in mismatch between the annual vaccine and circulating viruses. Low-resource countries remain mostly unprotected against seasonal influenza and are particularly vulnerable to future pandemics, in part, because investments in vaccine manufacturing and stockpiling are concentrated in high-resource countries. Antibodies that target conserved sites in the hemagglutinin stalk have been isolated from humans and shown to confer protection in animal models, suggesting that broadly protective immunity may be possible. Several innovative influenza vaccine candidates are currently in preclinical or early clinical development. New technologies include adjuvants, synthetic peptides, virus-like particles (VLPs), DNA vectors, messenger RNA, viral vectors, and attenuated or inactivated influenza viruses. Other approaches target the conserved exposed epitope of the surface exposed membrane matrix protein M2e. Well-conserved influenza proteins, such as nucleoprotein and matrix protein, are mainly targeted for developing strong cross-protective T cell responses. With multiple vaccine candidates moving along the testing and development pipeline, the field is steadily moving toward a product that is more potent, durable, and broadly protective than previously licensed vaccines.

## Highlights

• Current influenza vaccines are not broadly protective.
• Developing countries suffer the highest morbidity and mortality due to influenza.
• Correlates of protection and a regulatory pathway must be identified.
• Over 40 universal influenza vaccine candidates are currently in development.

Influenza viruses circulate globally and affect all age groups. Vaccines are the best tools available for preventing influenza illness and are recommended annually by the World Health Organization (WHO) for groups at high risk of mortality or significant morbidity, including pregnant women, children six months to five years of age, elderly individuals (65 years of age and older), individuals with chronic medical conditions, and health care workers. The US Centers for Disease Control and Prevention (CDC) have broadened the WHO recommendation for influenza vaccination to all persons older than six months of age, with two doses for children between six months and eight years old who are receiving influenza vaccine for the first time.

Human influenza viruses are classified into three types (A, B, C) based on the following highly conserved internal proteins: matrix protein 1 (M1), membrane matrix protein (M2), and nucleoprotein (NP). Type A influenza viruses are further sub-divided into subtypes based on the antigenicity of their hemagglutinin (HA) and neuraminidase (NA) surface glycoproteins. Currently, 18 HA and 9 NA subtypes of influenza A are known and all exist in aquatic birds, their natural reservoirs. Influenza B viruses infect only humans, but two antigenically and phylogenetically distinct lineages co-circulate. Type C influenza viruses are known to infect humans and pigs but infections are rare. Species tropism, particularly of influenza A, is determined by the presence, anatomic location and structure of terminal sialic acid residues that mediate viral attachment and entry. Influenza's antigenic variation, which has limited the development of a broadly protective vaccine, manifests as either antigenic drift or antigenic shift. Antigenic drift is the ability of the virus to escape pre-existing immunity through point mutations in the genes encoding HA and NA, making circulating strain prediction difficult and antigenic mismatch likely. Antigenic shift is defined by the recombination of HA genes that results in the generation of a novel influenza, a strain to which a large proportion of the human population has no pre-existing immunity, therefore increasing the potential for a pandemic, as occurred during the shift from H1 to H2 in 1957 and H2 to H3 in 1968.

The 2009 influenza A/H1N1 pandemic (a new strain of A/H1N1), the highly pathogenic avian influenza strains (A/H5N1 and A/H7N9), and past experiences with seasonal influenza vaccine mismatch have all illustrated the unpredictability of the virus and the challenges to mounting a global response against newly emerging virus strains. Currently, influenza A H1 and H3 subtypes co-circulate. The direct transmission of highly pathogenic avian strains to humans (such as influenza A/H5N1 and, more recently, influenza A/H7N9) presents an additional threat, but such strains have not yet demonstrated the capacity for efficient transmission in humans. Vaccination remains the preferred approach toward controlling influenza; however, the challenge of annual large-scale immunizations is currently beyond the capacities of low-resource countries, which need to improve vaccine delivery across all age groups, with a special focus on children. For such immunizations to become a reality, a vaccine that induces broadly cross-protective and durable immunity would need to be developed.

## 1 Licensed influenza vaccines and their limitations

**Fig. 1:**
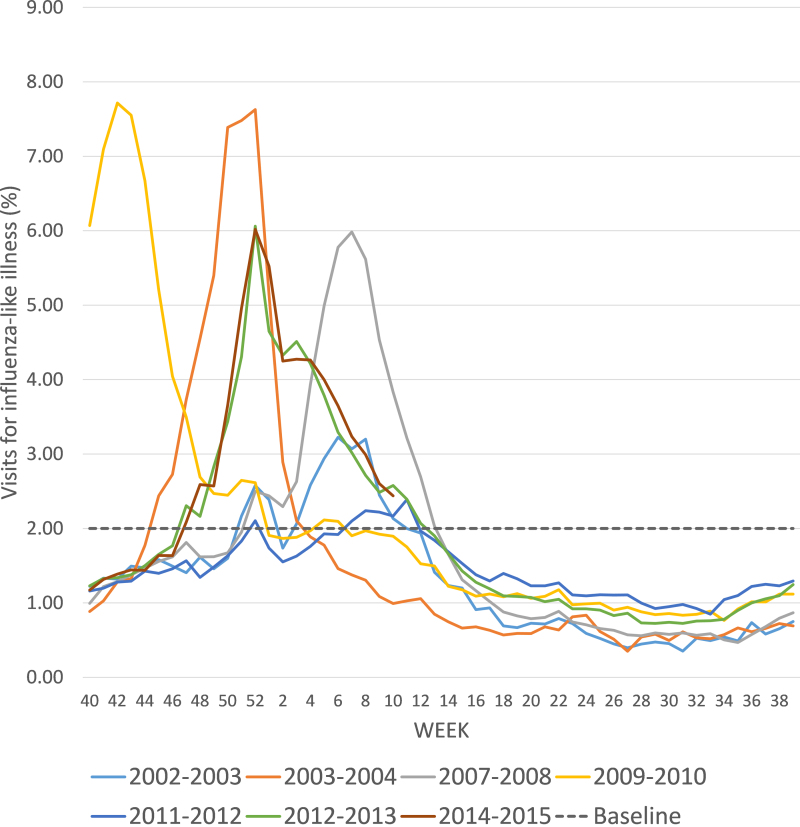
Percentage of patient visits for influenza-like illness in the US for selected seasons and years of mismatch. Scale represents percentage of patient visits for influenza-like illness for selected seasons discussed in the text. Data are from the US Centers for Disease Control and Prevention's US Outpatient Influenza-like Illness Surveillance Network. Data for 2014–2015 are through week 10, 2015. (For interpretation of the references to color in the citation of this figure, the reader is referred to the web version of this article.)

In general, influenza vaccines have an excellent safety record, but their efficacy varies significantly in various age groups and against different strains. Rare exceptions to the safety profile have emerged in association to the use of specific adjuvants, as discussed later. Strain mismatch and pandemics are frequent causes for vaccine failure and, over the last few years, several mismatches and one pandemic have occurred. The development and deployment of an H1N1 pdm2009 vaccine was relatively rapid, but the vaccine still became available too late to provide timely protection to vulnerable populations during the pandemic (Fig. 1, yellow line). Vaccine mismatch with seasonal influenza strains also has occurred multiple times, for example during the 2003/2004 season when an A/Fujian/411/2002 virus emerged as a new and unanticipated antigenic variant of influenza A/H3N2 (Fig. 1, orange line). Of 326 influenza A/H3N2 isolates characterized, only 25% were antigenically similar to the widely used vaccine strain A/Panama/2007/99 (H3N2). During the 2007/2008 influenza season, A/Wisconsin/67/2005 (H3N2) was included in the vaccine, but A/Brisbane/10/2007 (H3N2) was the dominant circulating strain, resulting in a particularly severe influenza season (Fig. 1, gray line). A mismatch of the vaccine to the recent 2012/2013 influenza season resulted in only 46% efficacy in adults 18 through 49 years and 9% efficacy in people older than 65 years of age (Fig. 1, green line). In this case, the circulating strain prediction was correct, but the mismatch was caused by a mutation in the egg-adapted seed virus (IVR-165) that was sent to vaccine manufacturers. The mutation was not present in the WHO-recommended strain but occurred during strain adaptation to growth in eggs.

Influenza vaccine mismatch also occurred during the 2014/2015 influenza season (Fig. 1, brown line). The influenza A/H3N2 strain included in the vaccine (A/Texas/50/2012) was selected based on the most common circulating influenza A/H3N2 virus in February 2014. The drifted influenza A/Switzerland/9715293/2013 virus was first detected in the United States in March 2014 in a small number of samples. Before the end of the year, it became the prevalent circulating strain and the vaccine available in the fall of 2014 offered poor protection against the mismatch, with an efficacy estimated around 18% for influenza A/H3N2. As a result, the influenza-associated hospitalization rate among people 65 years of age and older in the 2014/2015 season was the highest recorded since the CDC began tracking those data in 2005. In February 2015, WHO recommended that the new A/Switzerland/9715293/2013 (H3N2)-like virus to be included in the vaccine for the 2015/2016 season.

In the case of live-attenuated influenza vaccines (LAIVs), the efficacy of Flumist^®^ in randomized clinical trials was tempered by limited effectiveness against influenza A (H1N1) pdm09 in children during the 2013/2014 influenza season. These results may be specific to the influenza A/H1N1 component of the vaccine since the original A/California strain has been found to be more susceptible to thermal degradation due to a unique HA stalk sequence [1]. The full reason for its lack of effectiveness, however, is not entirely understood and is the subject of ongoing investigation. The past and current seasons exemplify the unpredictability of seasonal influenza epidemics and the challenge for current-generation vaccines that can only follow changes in circulating viruses. They also highlight opportunities for a universal vaccine that would induce broadly protective immunity.

## 2 General approaches for low- and middle-income country (LMIC) markets

While availability of data on disease burden in LIMIC remains limited, a recent study has shown that, during the year 2008, seasonal influenza was associated globally with 2–7% of deaths from acute lower respiratory infections (ALRI) in children younger than 5 years and resulted in 28,000–111,500 deaths, of which 99% occurred in developing countries. Influenza A, particularly H3N2 subtype, caused higher morbidity and mortality than influenza B [2]. As for pandemic outbreaks, models based on the 1918–1920 influenza pandemic estimate that up to 62 million people could die if a similar pandemic occurred today, and approximately 96% of the deaths would occur in the developing world [3]. Despite these figures, investments for improved seasonal and pandemic vaccine manufacturing and stockpiles are concentrated in high-resource countries. More than 80% of seasonal influenza vaccine doses produced between 2009 and 2010 originated from seven large manufacturers located in the United States, Canada, Australia, western Europe, Russia, China, and Japan. Moreover, no pandemic influenza A/H1N1 vaccine was available in the majority of low-resource countries before January 2010, more than eight months after the WHO declared a pandemic. Many low-resource countries do not have a full picture of their influenza disease burden due to limited surveillance. In some tropical settings, influenza circulates year-round, making influenza-related morbidity substantial, especially as a major contributor to childhood pneumonia [4].

Five companies currently offer WHO-prequalified split virion inactivated trivalent or quadrivalent seasonal influenza vaccines (GlaxoSmithKline, Green Cross Corp., Hualan Bio, Novartis, and Sanofi Pasteur) or seasonal trivalent LAIV (Serum Institute of India). Several companies (CSL, Green Cross Corp., MedImmune, Novartis, Sanofi Pasteur and Serum Institute of India) obtained prequalification of their pandemic influenza vaccines only for the 2009 pandemic influenza A/H1N1 virus. Despite sufficient influenza vaccine production capacity for annual needs in high-resource countries, influenza vaccination coverage rates among high-risk groups are below the targets set by national governments and recommended by WHO in these settings. In LMICs, differing health priorities and constraints on health budgets primarily limit influenza vaccine use to private markets, with the possible exception of Pan-American Health Organization countries. Expanding influenza vaccine coverage to public markets in LMICs is unlikely to happen in the absence of data from cost-benefit studies. Local production is expected to reduce cost and improve availability of vaccines in some LMICs, but the lack of local recommendations and government funding for vaccine use remains a limiting factor. Limited regional manufacturing capacity for seasonal vaccines also results in limited capacity to respond to pandemic outbreaks. The use of LAIVs may contribute to improved coverage rates in children since they cost less than IIVs to produce and administer, particularly if the age indication for current LAIV, currently two years of age, can be safely extended to a younger population.

## 3 Technical and regulatory assessment

Regulatory agencies recognize hemagglutination inhibition (HAI) titers as the primary correlate of protection and of influenza vaccine efficacy. In the case of LAIVs, the correlate of protection for universal vaccines might not be linked with hemagglutination inhibiting antibodies. Anti-stalk antibodies do not inhibit hemagglutination. Vaccine approaches based on viral proteins other than HA would also not affect hemagglutination. These factors pose a higher regulatory barrier. In the absence of an immune correlate, efficacy against a clinically meaningful endpoint will have to be demonstrated. The existing paradigm for currently licensed influenza vaccines is to define protection as the vaccine's ability to prevent illness associated with documented influenza infection. This is distinct from a vaccine's ability to reduce disease severity, which may be an important component of vaccine-mediated protection and may depend on both humoral and cellular immunity. Addressing a reduction in disease severity can be a worthwhile strategy in the development of universal or broadly reactive vaccines, but no influenza vaccine has been licensed so far on the basis of prevention of severe disease.

Despite the importance of animal models in the development of broadly reactive vaccines, carefully controlled human studies are essential. Field efficacy trials are costly and subject to a variety of confounding factors and biases. Human challenge studies allow for detailed measurements of the kinetics and magnitude of a range of immune responses, which can help with the development of correlates of protection. Wild-type influenza viruses have been used for challenge studies in adults, while attenuated vaccine strains of influenza have been used to perform challenge studies in children. In many instances however, the results of these studies have been difficult to interpret. In some instances, volunteers have been protected from influenza illness despite the lack of a measurable immune response to vaccination, although a possible explanation could be that disease was subclinical or not all non-vaccinated volunteers were reliably infected.

## 4 Status of influenza vaccine research and development activities

New technologies currently in development include faster generation of reassortant seed viruses, improved manufacturing consistency and yield, use of cell culture instead of eggs, improved adjuvants, quadrivalent vaccines, and prime-boost strategies. Other innovative approaches aim at inducing immune responses to less immuno-dominant epitopes, a phenomenon termed ‘unnatural immunity’ [5]. Vaccine candidates currently in the development pipeline (Table 1) can be divided into the following general categories: (1) those designed to elicit antibody responses to structurally conserved regions of HA and surface exposed membrane matrix protein (M2e), and (2) those that induce cross-protective T-cell responses against internal proteins like NP and M1. The former types of immunogens are intended to prevent infection while the latter are meant to reduce disease severity. Approaches targeting a cross-protective T-cell response against conserved internal influenza proteins such as NP and M1 are geared toward inducing a reduction in disease severity and could be valuable both for priming and boosting an immune response.

The HA molecule comprises two major structural elements, the head and stalk regions. The head is immuno-dominant and the primary target for currently licensed influenza vaccines. HAs are phylogenetically divided into group 1 (H1, H2, H5, H6, H8, H9, H11, H12, H13, H16, H17, and H18) and group 2 (H3, H4, H7, H10, H14, and H15) based on similarities in their stalk regions. With some exceptions, the head portion of the HA molecule is highly variable and protection by neutralizing antibodies targeting this region is limited to the same strain or subtype. On the other hand, monoclonal antibodies against conserved epitopes on the stalk recognize multiple subtypes. A murine monoclonal antibody, CR9114, has been shown to bind the HA stalk and protect against lethal challenge with influenza A and B [6]. Recently, a neutralizing antibody has been identified in humans that recognizes a conserved portion of the HA glycoprotein and neutralizes all 18 subtypes of both group 1 and group 2 influenza A viruses [7]. The challenge will be to develop an immunogen capable of inducing such antibodies.

Novel approaches have been undertaken to focus the immune responses against the HA stalk. In the first approach, a “headless” HA is designed by introducing a deletion in the HA sequence. Multiple approaches have recently been published to stabilize the headless stem. In one paper, researchers introduced mutations to stabilize the core of the hemagglutinin stem and bound these modified hemagglutinin to ferritin nanoparticles [8]. A second group applied a combination of mutations that realigned the subunits of the stem at the top resulting in soluble trimeric HA (mini-HAs) [9]. When the teams vaccinated mice, both groups saw full protection against H5N1. The nanoparticle-anchoring vaccine showed partial protection in ferrets, whereas the other vaccine showed partial protection in nonhuman primates. Future work will include extending these technologies to additional strains. Since viruses with a headless HA would be replication incompetent, this type of approach is limited to recombinant proteins, virus-like particles (VLPs), or nucleic acid vaccines.

The second approach used to overcome the sub-dominance of stalk epitopes is to induce anti-stalk antibodies via immunization with antigenically distant viruses. One strategy is to vaccinate sequentially with chimeric HA constructs having the same stalk but heads derived from different strains. Sequential exposure to the same stalk domain and divergent exotic head domains has been shown in animal models to boost anti-stalk cross-reactive antibodies. The induction of stalk-reactive antibodies has been demonstrated in humans after influenza A/H5N1 vaccine administration. Adults have pre-existing immunity against H1 (group 1 HA) and immunization with influenza A/H5N1 vaccine (also group 1 HA) induces high titers of stalk-reactive antibodies that are cross-protective in passive transfer experiments in mice [10].

Additional strategies to develop HA-based universal vaccines include Computationally Optimized Broadly Reactive Antigen (COBRA) from consensus HA sequences; HA peptides linked to the Ii-Key moiety of the class 2 Major Histocompatibility Complex (Antigen Express, Inc.); and fusion constructs with other proteins or immune-stimulant molecules (Table 1). Other influenza virus proteins could also be used to develop a universal vaccine. The influenza A virus ion channel M2 is expressed at high density on the cell membrane of virus-infected cells and at low density in the lipid membrane of the mature influenza virus. M2 has a small, non-glycosylated 24 amino acid ectodomain (M2e) that is highly conserved among human influenza A viruses. While natural infection does not usually induce an immune response to M2e in humans, antibodies can bind to M2e expressed on virus-infected host cells and reduce virus replication in experimental models. Since a robust M2e response might require the use of an adjuvant to induce high antibody titers, several M2e vaccine candidates are currently in clinical development as proteins conjugated to immune-stimulatory molecules to adjuvant the immune response [11]. Unfortunately, an M2e-based vaccine is unlikely to cover influenza B because its M2e comprises only four amino acids and is too small to be an effective immunogen.

Antibodies against NA interfere with virus release from the cell surface, effectively reducing the amount of infectious virus produced by infected cells. Two NA subtypes, N1 and N2, are commonly found in humans and NA, similar to HA, undergoes antigenic drift within a virus subtype. The majority of current seasonal vaccines include NA but its amount is not standardized, raising concerns that vaccines with low NA content may not induce NA antibodies at high enough titers to confer protection. Recent studies have shown that animals immunized with adjuvanted NA are protected against homologous and heterologous viral challenge, though within the same virus subtype [12]. Both NP and M1 proteins are well conserved in influenza A viruses. The stimulation of T cell-based immunity against those targets is considered an important aspect of the development of broadly reactive vaccines (Table 1). T cell-based immunity, however, is not expected to prevent infection, only to modulate it by reducing viral shedding and disease severity.

Adjuvants such as oil-in-water emulsions MF59 and ASO3 have been shown to increase the number of recognized HA epitopes and, consequently, to increase cross-recognition of non-matched strains. This was mainly demonstrated for pandemic influenza A/H5N1 vaccines which generally do not elicit robust cross-protection against multiple virus strains. MF59 in seasonal vaccines has thus far been well tolerated and has induced more potent, durable, and broader immune responses in children than non-adjuvanted vaccines. When evaluated in adult and elderly populations, MF59 has been shown to improve the immunogenicity of an influenza A/H5N1 vaccine. Notably, MF59 also boosted protection against influenza virus strains not fully matched to those included in the vaccine, as in the case of the A/Fujian/2002 H3N3 mismatch in 2003/2004 [13]. Other oil-in-water adjuvants used in influenza vaccines include AS03, although safety concerns have been raised after the increased incidence of narcolepsy and risk of anaphylaxis following administration of the 2009 AS03-adjuvanted monovalent pandemic A/H1N1 vaccine [14], [15].

## 5 Summary

No consensus has been achieved on what primary clinical endpoint universal influenza vaccines should achieve to be considered successful. Whether or not they should completely prevent influenza infection or reduce the severity of disease is still being debated. A truly universal influenza vaccine would offer a combination of protection from antigenic drift and shift and ideally confer lifelong immunity. Adjuvants have been moderately effective in increasing cross-reactivity against viral strains not included in the vaccine. Several novel technologies focused on innovative immunogens are currently in preclinical and early clinical development. While the influenza field has seen substantial investment over the last ten years, the majority of funding has focused on ensuring the sustainability of manufacturing within high resource settings and broadening manufacturing options so that influenza vaccines are not solely dependent on egg-based production. In recent years, however, investments have been increasing in the development of novel approaches to immunogen design, offering the potential for significant improvements in the performance of future influenza vaccines.

## Figures and Tables

**Table 1:** Development status of current vaccine candidates.

Organization	Approach, target, adjuvant	Preclinical	Phase 1	Phase 2	Phase 3	Licenced	Reference
Novartis (Switzerland)	Adjuvant MF59 allows for broader cross-reactivity against viral strains not included in the vaccine.				X	X	[13]
	Synthetic, self-amplifying mRNA, delivered by a synthetic lipid nanoparticle (SAM).	X					[16]
GlaxoSmithKline (UK)	Cross-clade antibody responses demonstrated with split-virion, inactivated, AS03 adjuvanted vaccine.				X	X	[17]
Icahn School of Medicine at Mount Sinai (USA) and GlaxoSmithKline (UK)	Various approaches to target conserved broadly reactive epitopes on HA stalk, such as ‘headless’ HA or functional chimeric HA (comprised of non-matched ‘head’ and ‘stalk’) expressed either in the context of whole virus or as rHA.	X					[18]
VaxInnate (USA)	Fusion protein between influenza M2e and bacterial flagellin (TLR5 ligand). Self-adjuvanted. Proposed to be used with conventional trivalent influenza vaccine (TIV).			X			[11]
Medicago (Canada)	Recombinant hemagglutinin (HA) expressed as virus-like particle (VLP) in tobacco plants. Requires adjuvant.			X			[19]
Immune Targeting Systems (UK)	Six long peptides from four core influenza proteins conjugated to fluorocarbon chain, elicits strong T cell response, proposed to be used with conventional TIV.			X			http://www.its-innovation.com
BiondVax Pharmaceuticals (Israel)	Multimeric-001 vaccine: recombinant protein, combination of nine conserved linear epitopes from HA, nucleoprotein (NP), and matrix protein (M).			X			[20]
SEEK (formerly PepTcell) (UK)	Flu-V: mixture of four chemically synthesized peptides targeting conserved T cell epitopes present in M1, NP, and M2 (with oil-in-water adjuvant).			X			[21]
Vivaldi Biosciences (USA and Austria)	Replication-Deficient Influenza virus created by deletion of the interferon-inhibiting NS1 protein activity.		X	X			[22]
Acambis Inc. (now Sanofi) (France)	ACAM-FLU-A Fusion between M2e and hepatitis B virus core protein (M2e-HBc) to produce VLPs presenting M2e.		X				[23]
Inovio (USA)	DNA plasmids encoding consensus sequences of HA, NA, and NP delivered by intradermal electroporation for eliciting antibody and T cell responses.		X				[24]
Dynavax (USA)	Fusion protein comprised of two highly conserved influenza antigens, NP, and M2e that are covalently linked to a proprietary immunostimulatory sequence.		X				http://investors.dynavax.com
Antigen Express (USA)	Synthetic peptides derived from conserved B cell epitopes from HA, linked to MHC Class 2 Ii-Key moiety for facilitated Th activity.		X				http://antigenexpress.com
National Institute of Allergy and Infectious Diseases (USA)	Fusion protein between self-assembling ferritin protein and full length HA for nanoparticle presentation of full length HA.	X					[8][26]
Crucell Vaccine Institute (The Netherlands) and The Scripps Research Institute (USA)	A stable trimeric influenza hemagglutinin stem (head-less) as a broadly protective immunogen (mini-HAs).	X					[9]
Jenner Institute, University of Oxford (UK)	Replication-deficient modified vaccinia virus Ankara (MVA) expressing both NP and M1. Designed for strong cross-reactive T cell response. Self-adjuvanted.	X	X				[25], [27]
	Replication-deficient simian adenovirus expressing both NP and M1. Designed for strong cross-reactive T cell response.	X	X				
	MVA expressing NP, M1, and conserved portion of HA.	X					
Cytos Biotechnology (Switzerland)	M2 protein linked to a TLR7 ligand yielding high levels of IgG2c antibodies.		X				[28]
Wistar Institute (USA)	Fusion protein between M2e and NP, expressed in chimpanzee adenovirus vector.	X					[29]
Gamma Vaccines (Australia)	Whole virion gamma-irradiated virus for intranasal application. Elicits B and T cell responses that are cross-protective. Self-adjuvanted.	X					[30]
Sanofi and VGTI (UK and USA)	VLP vaccine with computer optimized consensus HA sequence (COBRA—Computationally Optimized Broadly Reactive Antigen). Elicits broad antibody response. Alum adjuvanted.	X					[31]
FluGen (USA)	Single-replication influenza virus that is un-attenuated, but unable to shed. Designed to elicit humoral, mucosal, and cell mediated immunity (REDEE FLU).	X					http://flugen.com/redee-flu
University of Maryland, College Park (USA)	Rearranged genome of influenza virus permitting expression of two HA on the same virus while also being attenuated.	X					[32]
CureVac (Germany)	Synthetic mRNA encoding HA and NP. Temperature-stable product, elicits both B and T cell response, self-adjuvanted.	X					[33]
University of Pennsylvania (USA)	Adenovirus expressing broadly neutralizing monoclonal antibody against HA delivered by intranasal administration.	X					[34]
Georgia State University (USA)	Multiple M2 extracellular domains expressed in a VLP.	X					[35]
Merck Research Laboratories (USA)	Synthetic peptides of M2 extracellular domain conjugated to keyhole limpet hemocyanin or Neisseria meningitidis outer membrane protein complex.	X					[36]
Bionor (Norway)	Peptide-based approach targeting conserved epitopes (Vacc-Flu).	X					http://bionorpharma.com
VBI (formerly Variation Biotechnologies) (USA)	Unique technology using a mixture of 8 to 32 peptides, which represent hypervariable epitopes of HA to elicit polyclonal immune response.	X					http://www.vbivaccines.com
University of Wisconsin (USA)	Modified vaccinia virus Ankara encoding influenza virus HA and/or NP.	X					[37]
Codagenix, Inc. (USA)	Live attenuated influenza vaccine using Synthetic Attenuated Virus Engineering (SAVE1).	X					[38]
InvVax (USA)	Linear invariable epitopes used to construct non-variable influenza virus.	X					http://inv-vax.com
University Of Utah Research Foundation (USA)	Modified HA sequence with mutations that reduce antigenicity of immunodominant/variable epitopes.	X					WO2012082634
Okairòs (Italy, Switzerland)	Replication defective pan adenovirus type 3 vector expressing a fusion protein of M1 and NP.	X					[39]
University of Ghent (Vlaams Instituut voor Biotechnologie VIB) (Belgium)	Recombinant tetrameric protein, M2e-tGCN4 (modified form of the leucine zipper of the yeast transcription factor GCN4 linked to M2e).	X					[40]
University of Göteborg (Sweden)	Fusion protein based on the CTA1-DD adjuvant and containing tandem repeats of the M2e ectodomain epitope.	X					[41]
Tsinghua University (China)	Synthetic peptide (N-terminus of M2e) coupled to carrier protein.	X					[42]
University of Ottawa (Canada) and National Institutes for Food and Drug Control (China)	Adenovirus vaccine encoding secreted fusion protein (codon-optimized HA2 subunit fused to a trimerized form of murine CD40L).	X					[43]
California Institute of Technology (USA)	Adeno-associated viruses delivered intramuscularly encoding two broadly neutralizing antibodies.	X					[44]
